# Clinical and Cytometric Study of Immune Involvement in a Heterogeneous Cohort of Subjects With RASopathies and mTORopathies

**DOI:** 10.3389/fped.2021.703613

**Published:** 2021-08-13

**Authors:** Erica Valencic, Prisca Da Lozzo, Gianluca Tornese, Elena Ghirigato, Francesco Facca, Elisa Piscianz, Flavio Faletra, Andrea Taddio, Alberto Tommasini, Andrea Magnolato

**Affiliations:** ^1^Department of Pediatrics, Institute for Maternal and Child Health (IRCCS) “Burlo Garofolo”, Trieste, Italy; ^2^Department of Medical, Surgical and Health Sciences, University of Trieste, Trieste, Italy; ^3^Department of Diagnostics, Institute for Maternal and Child Health (IRCCS) “Burlo Garofolo”, Trieste, Italy

**Keywords:** RASopathies, mTORopathies, Ras/MAPK, recent bone marrow emigrants, flow cytometry, immune dysregulation

## Abstract

RASopathies and mTORopathies are groups of genetic syndromes associated with increased activation of the RAS-MAPK or the PI3K-AKT-mTOR pathway, resulting in altered cell proliferation during embryonic and postnatal development. The RAS-MAPK and the PI3K-AKT-mTOR pathways are connected to each other and play a crucial role in adaptive immunity. However, with the exception of Activated PI3K delta syndrome (APDS), immune function has not been deeply studied in these disorders. We collected clinical and immunophenotypic data of a cohort of patients with RASopathies and mTORopathies. Overall, we enrolled 47 patients (22 females, 25 males, age 2–40 years): 33 with neurofibromatosis type 1, 11 Noonan syndrome and 3 Bannayan-Riley-Ruvalcaba syndrome. 8 patients reported a history of invasive infections requiring hospitalization and intravenous antibiotic therapy. Only 3 patients reported a history of unusual, difficult-to-treat or deep-seated infection. Adenotonsillectomy was performed in 11 patients (24%). However, in most cases (83%) patients' parents did not perceive their child as more prone to infections than their peers. Lymphocyte subpopulations were analyzed in 37 of the 47 patients (16 female, 21 males, age 1–40 years). Among the studied lymphocyte subsets, the only consistent alteration regarded an increased percentage of immature B cells (recent bone marrow emigrants) in 34 out of 37 (91,9%) patients, and an increased percentage of double negative T cells in 9 patients. In conclusion, although borderline immune abnormalities were present in a significant proportion of subjects and adenotonsillectomy was performed more frequently than expected for the general population, no major immune disturbance was found in this cohort of patients.

## Introduction

RASopathies are a class of genetic syndromes due to germline mutations in genes that encode protein components of the RAS-mitogen activated protein kinase (MAPK) pathway (1). This wide group includes Neurofibromatosis type 1 (NF1), Noonan syndrome (NS), Noonan syndrome with multiple lentigines (i.e. LEOPARD syndrome), cardiofaciocutaneous (CFC) syndrome, Costello syndrome, Legius syndrome and capillary malformation-arterovenous malformation (CM-AVM) syndrome. Although each syndrome exhibits distinctive clinical features, these disorders share significant overlapping characteristics that include short stature, facial dysmorphisms, congenital cardiac defects, skin lesions, varying degrees of neurocognitive impairment, and increased risk of malignancies. The overall prevalence averages approximately 1 in 1,000 live births (1, 2). All the mutations found in RASopathies result in increased signaling and/or constitutive activation of the RAS-MAPK pathway, thus impacting proliferation of many cell types during embryonic and postnatal development (1). Activating mutations of the RAS-MAPK pathway genes are associated with oncogenic transformation and carcinogenesis, with RAS found to be somatically mutated in 20% of malignancies (3, 4).

Moreover, RAS plays an essential role in adaptive immunity and normal immune cell function. Evidence shows that RAS is rapidly activated in antigen and cytokine receptor-stimulated lymphocytes, resulting in increased cytokine expression and development of T and B cells (5).

Following activation, RAS-GTP conveys the extracellular signal through three primary effector arms, the RAF-MEK-ERK pathway, the PI3K-AKT pathway, and the RalGDS signaling pathway.

**Figure 1:**
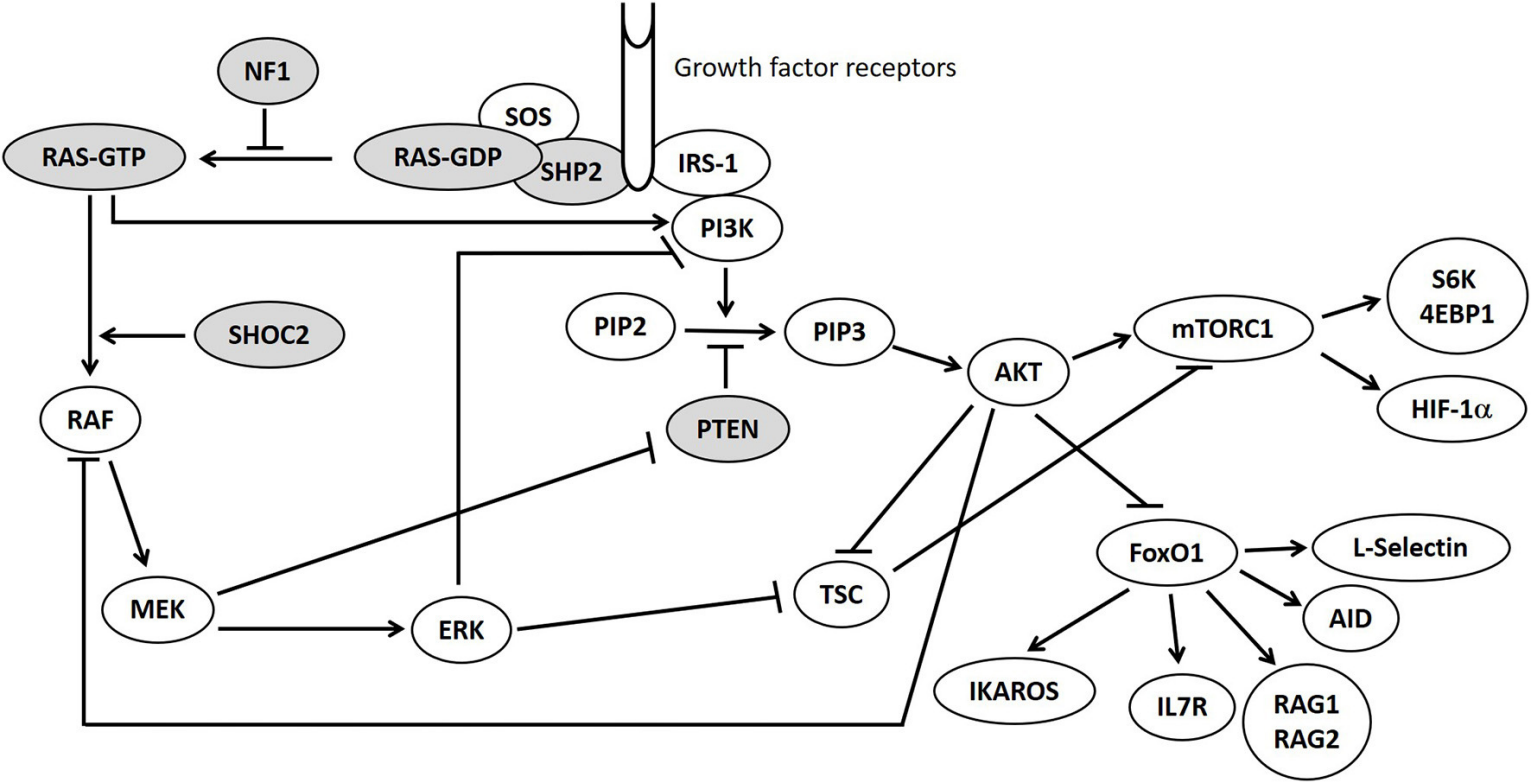
RAS/MAPK and PI3K-AKT-mTOR pathways. Mutations in genes displayed in gray circles are responsible for Neurofibromatosis type 1 (NF1), Bannayan-Riley-Ruvalcaba syndrome (PTEN) and Noonan syndrome (PTPN11, KRAS, SHOC2) in our patients.

The RAS-MAPK pathway is therefore closely connected to the PI3K-AKT-mTOR signaling pathway and they are both involved in the regulation of mTOR and FOXO1 (Figure 1), which are crucial regulators of cells proliferation and immune homeostasis (6). Mutations in proteins belonging to PI3K-AKT-mTOR pathway lead to various syndromes (“mTORopathies”) including Bannayan-Riley-Ruvalcaba syndrome (BRRS), tuberous sclerosis, Peutz-Jeghers syndrome characterized by hyperactivation of mTOR-signaling (7).

Dysregulation of PI3K-AKT pathway was recently discovered to be causative of a rare monogenic immunodeficiency called Activated PI3 kinase Delta Syndrome (APDS) (8). The shared hyperactivation of PI3K-AKT-mTOR pathway may account for some degree of immune imbalance in RASopathies and mTORopathies similarly to APDS. However, little is known about the consequences of RAS-MAPK and PI3K-AKT-mTOR dysregulation pathways seen in these distinct syndromes on the immune cell homeostasis and functions. Although serious immune disturbances have not been reported, no study directly focused on clinical symptoms and immunological signs of immunodeficiency in these patients.

With the present study we aimed to analyze the clinical susceptibility to infections and the immune phenotype in patients with RASopathy and mTORopathies.

## Materials and Methods

We recruited consecutive patients referred to the IRCCS Burlo Garofolo at Rare Disease Service because of routine follow up from October 2016 to September 2020 with a diagnosis of a RASopathy or mTORopathy, namely Neurofibromatosis type 1, Noonan syndrome, Noonan syndrome with multiple lentigines, cardiofaciocutaneous syndrome, Costello syndrome, Legius syndrome, capillary malformation-arteriovenous malformation syndrome and Bannayan-Riley-Ruvalcaba syndrome. Informed consent was obtained from all participating subjects.

Inclusion criteria were: patients with a diagnosis of any of the group of disorders known as RASopathies or mTORopathies, as listed above, made clinically and/or confirmed through genetic testing. Exclusion Criteria were: patients who did not have the ability/capacity to undergo the informed consent process or whose parent/legal guardian was unable to undergo the informed consent process; lack of available clinical data.

Experimental design of the study consists of two parts: in the first part clinical information about infections (frequency, severity, duration, and type), lymphoproliferation was collected for each patient from the Rare Diseases Unit database of the “Burlo Garofolo” Institute for Maternal and Child Health.

Moreover, a structured non-validated anamnestic questionnaire about frequency, type and severity of past infections, past surgeries, history of lymphadenopathy or splenomegaly was offered to the patients' families; one question specifically asked about the parents' perspective of the immune status of their children in terms of susceptibility to infections (Supplementary Table 1). In the second part of the study, laboratory work-up was performed on patients who returned for clinical examination during the enrollment period and were submitted to blood withdrawal for other diagnostic tests.

Peripheral blood was collected from patients and healthy donors and processed within 24 hours. Multicolor immunophenotyping was assessed in order to evaluate recent thymic emigrants (RTE, defined by CD3, CD4, CD31 and CD45RA expression), CD4/CD8 ratio, double negative T cells (DNT, defined as CD3+CD4-CD8-TCRα/β+), transitional B cells/recent bone marrow emigrants (RBE, defined as as CD19+IgD/IgM+CD10+), IgM B memory cells (Bmem, defined as CD19+IgD/IgM+CD27+) and switched memory B cells (Bswi, defined as CD19+IgD/IgM-CD27+). Three antibodies panels were used: (1) CD3 VioBlue, CD45 VioGreen, CD16 Viobright515, CD56 Viobright515, CD31 PE, CD8 PerCP-Cy5.5, CD45RA PE-Vio770, CD4 APC, CD19 APC-Cy7; (2) CD3 VioBlue, CD45 VioGreen, TCRγ/δ FITC, TCRα/β PE, CD8 PerCP-Cy5.5, B220 PE-Vio770, CD4 APC, CD95 APC-Vio770; (3) CD21 VioBlue, CD45 VioGreen, CD38 FITC, IgD PE, IgM PE, IgG PerCP-Vio700, CD10 PE-Vio770, CD27 APC, CD19 APC-Cy7.

Samples were acquired with MACSQuant Analyzer 10 or BD FACSCalibur cytometers and analyzed with FlowLogic software. Reference values for lymphocytes subpopulations have been derived and adapted from van Gent et al. (9); results out of these ranges are considered abnormal.

Since several multiparameter flow cytometric assays have been suggested to characterize RBE subpopulation and normal ranges change on the basis of markers evaluated, we also measured this population in 18 age-matched healthy donors. Children undergoing elective surgery procedures and residents were considered to be a control group.

Statistical analysis on RBE data was performed using one way analysis of variance (ANOVA) and Holm-Sidak's post-test correction in the case of multiple comparisons. Analysis was performed using GraphPad Prism software (version 7.0).

## Results

Clinical data from 47 patients with RASopathy and mTORopathies were analyzed (Supplementary Tables 2, 3). The cohort comprised 33 patients affected with Neurofibromatosis type 1, 11 patients with Noonan syndrome and 3 patients with Bannayan-Riley-Ruvalcaba syndrome. Gender was equally distributed, with 22 females and 25 males. Patients' age ranged from 2 to 4054 years; 35 patients (75%) were below 18 years of age.

8 patients (17%) had a history of invasive infections requiring intravenous antibiotic therapy, 10 (21%) reported no past history of infection requiring antibiotic therapy, 29 (62%) patients reported usually one, or less than one, infection requiring oral antibiotics in a year, 6 patients reported more than one infection in a year. 40 (85%) reported no infection requiring antibiotics (oral or intravenous) in the last year. The most common infections were pneumonia among the invasive cases and upper respiratory infections among the milder cases (requiring oral antibiotics). Only 3 patients reported a history of unusual or difficult-to-treat infections.

67% of the study population underwent a surgical procedure during life, in 11 patients (24%) adenotonsillectomy was performed, 8 patients (17%) underwent genitourinary surgical procedure, 6 patients underwent surgical procedure for skin issues.

In 39 cases (83%) patients' parents did not perceive their child as more prone to infections in comparison with their peers.

Lymphocyte subpopulations analysis was subsequently carried out in 37 of 47 enrolled patients (16 female, 21 males) (Supplementary Table 2). This cohort of patients comprised 23 patients affected with NF1, 11 patients with NS, and 3 patients with BRRS (age range 2–40 years).

**Figure 2:**
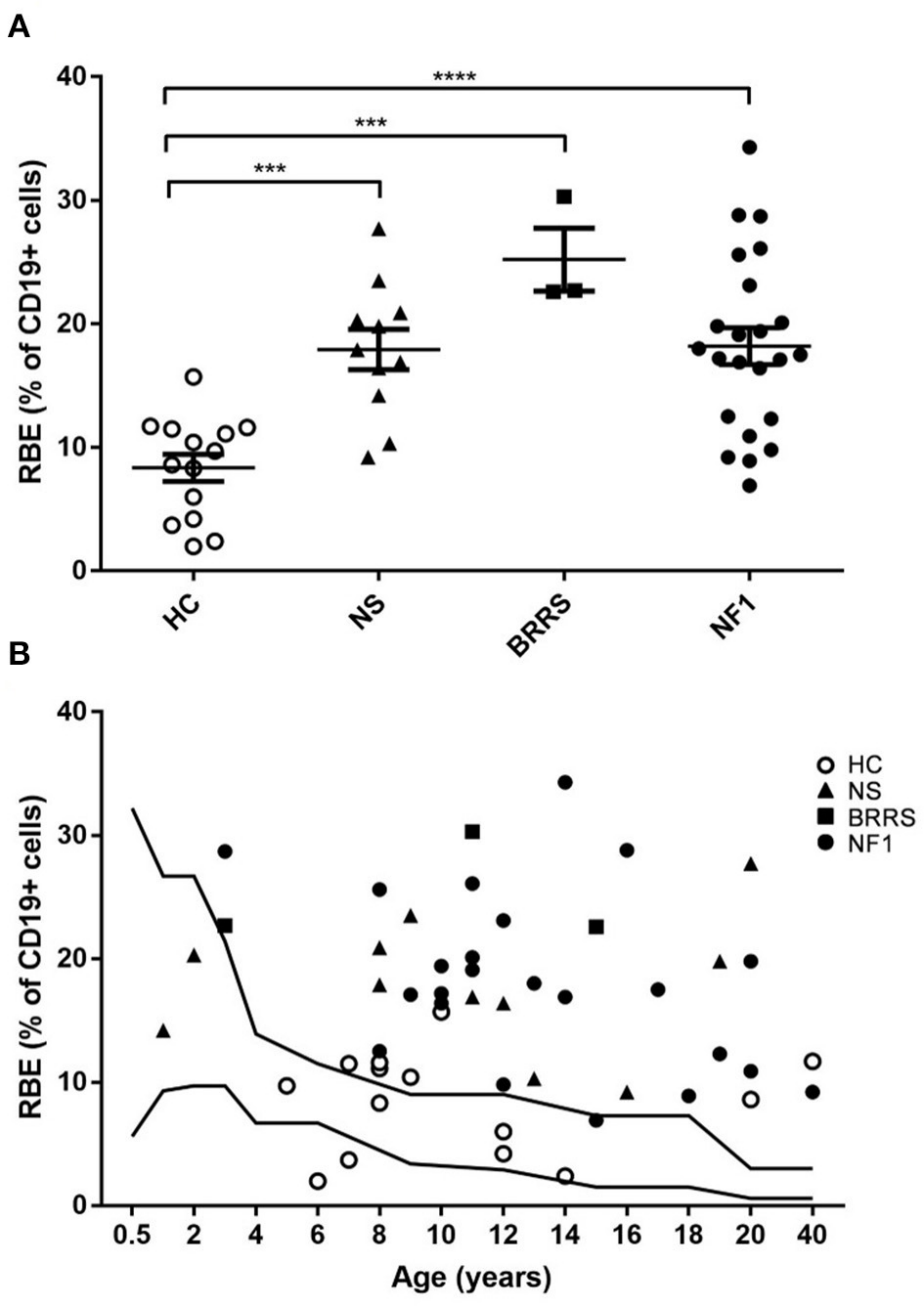
Recent bone marrow emigrants (RBE) values by flow cytometry. **(A)** RBE expressed as percentage of lymphocytes B (CD19+ cells) grouped according to healthy controls and to the three syndromes evaluated in our cohort. Statistical analysis was performed using one way ANOVA multiple comparisons (Holm-Sidak's post-test correction); only statistically significant findings are highlighted in figure. *** *P* < 0.001; **** *P* < 0.0001. **(B)** RBE expressed as percentage of lymphocytes B (CD19+ cells) displayed according to age. HC, healthy controls; NS, Noonan syndrome; BRRS, Bannayan-Riley-Ruvalcaba syndrome; NF1, Neurofibromatosis type 1.

Data indicated that RBE frequency was above the normal range standardized for age in 34 out of 37 (91,9%) patients; only 2 two-year-old babies affected with NS and a girl affected with NF1 showed normal values of RBE (Figure 2).

Analysis of the mature compartment of B cells showed that frequency values of IgM memory B cells (Bmem) and switched memory B cells (Bswi) were within the reference range in most of patients: both values were found below the lower limit of the reference range in one patient affected with BRRS; another three patients affected with NS had low values of Bmem or Bswi cells.

**Figure 3:**
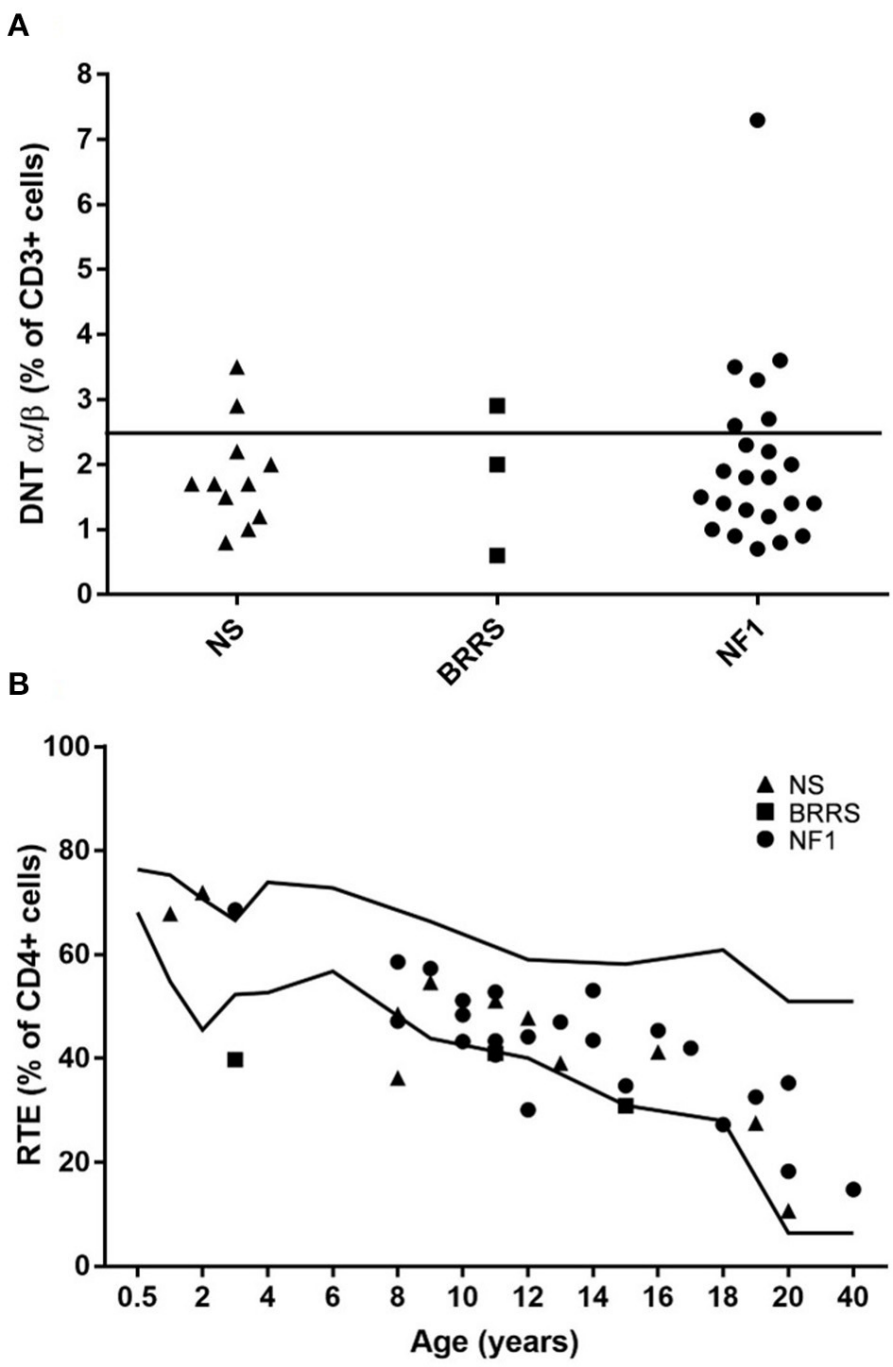
**(A)** Double negative T (DNT) α/β+ cells expressed as percentage of lymphocytes T (CD3+ cells) grouped according to the three syndromes evaluated in our cohort. **(B)** Recent thymic emigrants (RTE) expressed as percentage of lymphocytes T helper (CD4+ cells) displayed according to age. NS, Noonan syndrome; BRRS, Bannayan-Riley-Ruvalcaba syndrome; NF1, Neurofibromatosis type 1.

Only 3 patients had a low percentage of the RTE population, below the reference values. In 9 patients DNT population was higher than 2, 5 % of CD3 lymphocytes, which is the threshold used in the screening of autoimmune lymphoproliferative disorders (Figure 3).

Finally, CD4/CD8 ratio was inverted in 2 patients affected with NF1.

## Discussion

The RAS-MAPK pathway is responsible for the signal transduction from the cell surface to the nucleus in response to specific growth factors and is essential in the regulation of cell cycle, differentiation, growth and cell senescence, all of which are important for normal development and cell homeostasis.

The importance of the RAS-MAPK pathway in adaptive immunity and normal immune cell function is well established but the implications on immune status of patients with RASopathies and their immune phenotype have not been specifically investigated. Hyperactivating mutations in hRAS and nRAS have been associated with lymphoproliferation and risk of developing Juvenile Myelomonocytic Leukemia (10). Interestingly, the RAS-MAPK pathway is closely connected with the PI3K-AKT-mTOR signaling, which has a crucial role in regulating immune cell proliferation and differentiation. Indeed, mutations in *PIK3CD* or *PIK3R1*, resulting in hyperactive PI3K-AKT-mTOR, have also been associated with lymphoproliferation and defective lymphocyte development (Activated PI3 kinase delta syndrome, APDS). APDS can manifest with recurrent bacterial respiratory infections, bronchiectasis, severe, persistent or recurrent herpes virus infections, lymphadenopathy, splenomegaly, autoimmune or autoinflammatory manifestations, neurodevelopmental delay, defective IgG production and increased lymphoma susceptibility. Moreover, we showed that subjects with a PIK3R1 deficiency have a defective B cell maturation in the early stages with an increase in RBE, probably due to reduced FOXO1 activity (11).

In this context, we postulated that patients with other RASopathies and mTORopathies might express some degree of clinical or subclinical immune dysregulation because of impaired control of the RAS-MAPK and PI3K-AKT-mTOR pathways.

In 2016 Torres et al. reported no differences between NF1 patients and healthy controls in their frequencies of CD8+ T cells, CD56+ NK cells, CD14+ monocytes and CD14+/CD16+ monocytes. In addition, no significant difference was found in the frequency of CD19+ B lymphocytes, although there was a very significant decrease in CD19+ B cells expressing CD5+ in NF1 patients (12). However, these works did not investigate specific lymphocyte subpopulations whose percentages are typically altered in APDS or RALD, such as RBE, switched memory B cells, recent thymic emigrants (RTE) and double negative CD4-CD8- T cells with alpha/beta receptor (DNT) (11, 13). Furthermore, these studies did not assess the possible presence of minor clinical signs that could arise from imbalanced immune homeostasis, like recurrent or serious infections and lymphoproliferative features.

We showed that patients with RASopathies do not display significant clinical signs of immunodeficiency in terms of recurrent, deep-seated, difficult-to-treat or unusual infections. However, lymphocyte immunophenotyping revealed that almost all patients had higher than expected percentages of B cells of recent bone marrow derivation (RBE), similarly to what is known in APDS. Even if there was no significant change in other B cell subsets associated with immunodeficiency, it is possible to hypothesize that some degree of delay in B-cell maturation is present also in these patients. However, the risk of infection in subjects with APDS and more in general with common variable immunodeficiency relies on low percentages of switched memory B cells and reduced RTE, which were normal or just slightly reduced in our cohort. This may explain why the risk of infections seemed not increased in our series. Conversely, it is possible that such mild perturbance of B cell maturation could be related with a trend for tonsillar and adenoid hypertrophy in patients with RASopathies and mTORpathies, since B cells are a prevalent lymphocyte population in such organs. Interestingly, 24% of the patients in our series underwent adenotonsillectomy, suggesting that adenoid and tonsillar hypertrophy might represent a minor clinic sign of lymphoproliferation associated with the underlying disorders. In fact, although the prevalence of adenotonsillectomy in a matched population is not known, according to recent data the rate of tonsillectomy in the general population in Italy, averaging 0.5/1000 inhabitants/year, is too low to yield a prevalence higher than 5–10% in a lifespan^1^ Since this study is retrospective and the collection of clinical data is based on patients' interviews, we cannot be confident that all adenotonsillectomy procedures had been performed because of lymphoproliferative signs. However, we previously showed that a treatment with sirolimus could both cure autoimmune cytopenias and reduce lymphoproliferation in subjects with ALPS and in a young girl it was even associated with shrinking of adenoids and resolution of recurrent otitis (14). Considered that sirolimus may have other beneficial effects in subjects with RASopathies and mTORopathies, it could be intriguing to know if such a treatment could also reflect on a better control of signs arising from tonsil or adenoid hypertrophy (15). Furthemore, data regarding elevated DNT in a subgroup of patients of our cohort may also suggest a relationship with other disorders associated with lymphoproliferation and impaired immune homeostasis, such as RAS-associated Autoimmune Lymphoproliferative disorder (RALD) and Autoimmune Lymphoproliferative Syndrome (ALPS). Of note, both RALD and ALPS can be associated with an activated PI3K-AKT-mTOR pathway (16–18). These immune disorders are characterized by persistent non-malignant and non-infectious lymphadenopathy and/or splenomegaly, autoimmune cytopenias and an increase of circulating DNT cells (>3% of T cells). In our cohort, the incidence of autoimmune disorder was not increased, as autoimmune thyroiditis was present only in one case. However, previous studies suggested that the risk of autoimmune phenomena in RASopathies tend to increase with age, whilst the age in our series averaged 13 years (19).

## Data Availability Statement

The raw data supporting the conclusions of this article will be made available by the authors, without undue reservation.

## Ethics Statement

The studies involving human participants were reviewed and approved by CEUR Friuli Venezia Giulia, project RC 24/17. Written informed consent to participate in this study was provided by the participants' legal guardian/next of kin.

## Author Contributions

EV: Conceptualization and writing. PD: Writing. GT and AM: Patient enrollment. EG and FFac: Clinical database creation. EP: Investigations. FFal: Genetic counselling. ATa: Review and editing. ATo: Funding acquisition. AM: Supervision. All authors have read and agreed to the published version of the manuscript.

## Conflict of Interest

The authors declare that the research was conducted in the absence of any commercial or financial relationships that could be construed as a potential conflict of interest.

## Publisher's Note

All claims expressed in this article are solely those of the authors and do not necessarily represent those of their affiliated organizations, or those of the publisher, the editors and the reviewers. Any product that may be evaluated in this article, or claim that may be made by its manufacturer, is not guaranteed or endorsed by the publisher.

## Footnotes

^1^https://ec.europa.eu/eurostat/statistics-explained/index.php?title=Surgical_operations_and_procedures_statistics.
