# Supplementary material for: Clinical and Cytometric Study of Immune Involvement in a Heterogeneous Cohort of Subjects With RASopathies and mTORopathies
Source: Front Pediatr. 2021 Aug 13;9:703613. doi: 10.3389/fped.2021.703613 (PMC8414575; doi:10.3389/fped.2021.703613)
Supplement: Supplementary file 1 [file Data_Sheet_1.docx]

Supplementary Material

**Supplementary Table 1.** English translation of the questionnaire administered to the patients

| 1. Did your son/daughter ever need hospital admission for treatment with intravenous antibiotics because of an infection? | | | |
| --- | --- | --- | --- |
| a. | Never | |  |
| b. | Once | |  |
| c. | More times | |  |
|  |  | - Less than once a year | |
|  |  | - Two or more times a year | |
| 2. How many times did your son/daughter need oral antibiotics for non-serious infections? | | | |
| a. | Never | |  |
| b. | Once | |  |
| c. | More times | |  |
|  |  | - Less than once a year | |
|  |  | - Two or more times a year | |
| 3. If he/she had serious infections, what was the site? (Check all that applies) | | | |
| a. | Systemic (sepsis/meningitis) | |  |
| b. | Upper respiratory tract (ear, nose and throat) | |  |
| c. | Lower respiratory tract (pneumonia) | |  |
| d. | Gastrointestinal | |  |
| e. | Bones | |  |
| f. | Kidney | |  |
| g. | Skin | |  |
| h. | Mouth and teeth | |  |
| 4. If he/she had non-serious infections requiring antibiotics, what was the site? (Check all that applies) | | | |
| a. | Systemic (sepsis/meningitis) | |  |
| b. | Upper respiratory tract (ear, nose and throat) | |  |
| c. | Lower respiratory tract (pneumonia) | |  |
| d. | Gastrointestinal | |  |
| e. | Bones | |  |
| f. | Kidney | |  |
| g. | Skin | |  |
| h. | Mouth and teeth | |  |

| 5. Did your son/daughter undergo surgical procedures? If yes, which ones? | | | | |
| --- | --- | --- | --- | --- |
| a. | No procedure | |  | |
| b. | Yes | |  | |
|  |  | - Appendicectomy | | |
|  |  | - Adenoidectomy/tonsillectomy | | |
|  |  | - Other (please, specify) | | |
| 6. How often has your son/daughter gotten sick with infections not requiring antibiotic therapy so far? | | | | |
| a. | Never | |  | |
| b. | Once | |  | |
| c. | 2-4 times | |  | |
| d. | 5-8 times | |  | |
| e. | >9 times | |  | |
| 7. In the last year, how many times has your son/daughter gotten sick with infections treated with antibiotics? | | | | |
| a. | Never | |  | |
| b. | Once | |  | |
| c. | 2-4 times | |  | |
| d. | 5-8 times | |  | |
| e. | >9 times | |  | |
| 8. Does one of the following conditions apply to your son/daughter? | | | | |
| a. | Four or more episodes of acute otitis media in the last year | | |  |
| b. | Two or more episodes of sinusitis in the last year | | |  |
| c. | Two or more episodes of pneumonia in the last year | | |  |
| d. | Any infection that doctors considered unusual (unusual pathogen, severe course) | | |  |
| e. | Recurrent infections caused by the same bacteria | | |  |
| f. | Severe clinical course after common trivial infection | | |  |
| g. | Need for prolonged treatment with antibiotics because of a protracted infection | | |  |
| h. | No one of the above | | |  |
| 9. Do you feel that your son/daughter is more prone to infections than his/her peers? | | | | |
| a. | Yes | |  | |
| b. | No | |  | |
| 10. Has your son/daughter ever gotten sick with mononucleosis? | | | | |
| a. | Yes | |  | |
|  |  | - He/she was admitted to hospital because of it | | |
|  |  | - He/she was treated at home | | |
| b. | No | |  | |

| 11. Did your son/daughter ever complain of swelling of lymph nodes in neck or in other sites? | | | |
| --- | --- | --- | --- |
| a. | Yes | |  |
|  |  | - Please, specify the site/s | |
| b. | No | |  |
| 12. Did your son/daughter ever complain of medical conditions involving the liver or the spleen? | | | |
| a. | Yes | |  |
|  |  | - Please, specify the site/s | |
| b. | No | |  |

**Supplementary Table 2.** Baseline demographic and clinical characteristics of the study population.

|  | Genetics | Age (y) | Cafè-au-lait spots | Freckling | Skin neurofibroma | PleXiform neurofibroma | Xanthogranuloma | Pigmentid macules of penis | Intestinal hamartomatous polyposis | Lipomas | macrocephaly | Short stature | Cognitive retardation | learning and/or behavioral problems | Congenital heart defects | Splenomegaly | Lymphadenopathy | Eye anomalies | Skeletal anomalies | Genitourinary disorders | SNC anomalies | Pulmonary anomalies | Autoimmunity | Deafness | RBE | DNT | RTE |
| --- | --- | --- | --- | --- | --- | --- | --- | --- | --- | --- | --- | --- | --- | --- | --- | --- | --- | --- | --- | --- | --- | --- | --- | --- | --- | --- | --- |
| 1 | NF1 | 2 | X | X |  |  |  |  |  |  | X |  |  |  |  |  |  |  |  |  |  |  |  |  | ND | ND | ND |
| 2 | NF1 | 5 | X | X |  |  |  |  |  |  | X |  | X |  |  | X | X |  |  | X |  |  |  |  | ND | ND | ND |
| 3 | NF1 | 5 | X | X | X |  |  |  |  |  | X |  | X |  |  | X |  |  |  | X |  |  |  |  | ND | ND | ND |
| 4 | NF1 | 29 | X | X | X | X |  |  |  |  | X |  |  |  |  |  |  |  |  |  |  |  |  |  | **10.9** | 1.0 | 18.3 |
| 5 | NF1 | 16 | X | X |  |  |  |  |  |  |  |  |  |  |  | X |  |  |  |  |  |  |  |  | 6.9 | 0.9 | 34.8 |
| 6 | NF1 | 11 | X | X |  |  |  |  |  |  |  |  |  |  |  |  |  |  |  |  |  |  |  |  | **19.1** | 1.8 | 40.7 |
| 7 | PTEN | 15 | X |  |  |  |  | X | X | X | X |  | X |  |  |  |  |  |  |  |  |  |  |  | **22.6** | 0.6 | 30.9 |
| 8 | NF1 | 11 | X | X | X | X |  |  |  |  |  |  |  |  |  |  |  |  |  |  | X |  |  |  | **16.4** | 1.2 | 43.3 |
| 9 | NF1 | 32 | X | X | X | X |  |  |  |  |  |  |  |  |  |  |  |  | X | X |  |  | X | X | ND | ND | ND |
| 10 | Neg | 13 | X | X | X |  |  |  |  |  | X |  |  |  |  |  |  |  |  |  |  |  |  |  | **23.1** | 1.4 | **30.1** |
| 11 | NF1 | 10 | X | X | X |  |  |  |  |  |  |  |  | X |  |  |  |  | X |  |  |  |  |  | **19.4** | 2.0 | 51.2 |
| 12 | NF1 | 11 | X | X | X |  |  |  |  |  | X |  |  | X |  |  |  | X |  |  |  |  |  |  | **17.2** | 1.5 | 48.4 |
| 13 | NF1 | 41 | X | X | X | X |  |  |  |  |  |  |  |  |  |  |  |  |  | X | X |  |  |  | **9.2** | 1.3 | 14.8 |
| 14 | Neg | 8 | X | X |  |  |  |  |  |  | X |  |  |  |  |  |  |  |  |  |  |  |  |  | **12.5** | **7.3** | 58.6 |
| 15 | PTPN11 | 16 |  |  |  |  |  |  |  |  |  | X |  |  | X |  |  |  |  |  |  |  |  |  | **9.2** | 1.0 | 41.2 |
| 16 | NF1 | 2.5 | X | X | X | X |  |  |  |  | X |  |  |  |  |  |  |  |  |  | X |  |  |  | ND | ND | ND |
| 17 | Neg | 11 | X | X |  |  |  |  |  |  |  | X | X | X | X |  |  |  |  |  |  |  |  |  | ND | ND | ND |
| 18 | Neg | 10 | X | X |  |  |  |  |  |  | X |  |  |  |  |  |  |  |  |  |  |  |  |  | ND | ND | ND |
| 19 | Neg | 12 | X | X |  |  |  |  |  |  |  |  |  |  |  |  |  |  |  |  |  |  |  |  | **26.1** | 2.3 | 52.8 |
| 20 | Neg | 8 | X | X |  |  | X |  |  |  |  |  |  |  |  |  |  |  |  |  |  |  |  |  | ND | ND | ND |
| 21 | NF1 | 18 | X | X |  |  |  |  |  |  |  |  |  |  |  |  |  |  |  |  |  |  |  |  | **8.9** | **2.6** | 27.3 |
| 22 | NF1 | 12 | X | X | X |  | X |  |  |  |  |  |  |  |  |  |  |  |  |  |  |  |  |  | ND | ND | ND |
| 23 | NF1 | 37 |  |  |  |  |  |  |  |  |  |  |  |  |  |  |  |  |  |  |  |  |  |  | ND | ND | ND |
| 24 | PTPN11 | 12 |  |  |  |  |  |  |  |  |  | X |  | X | X |  |  | X | X | X |  |  |  |  | **16.4** | 2.2 | 47.8 |
| 25 | NF1 | 13 | X | X | X |  |  |  |  |  |  |  |  |  |  |  |  |  |  |  |  |  |  |  | **18.0** | 0.7 | 47.0 |
| 26 | NF1 | 16 | X | X |  |  |  |  |  |  |  |  |  | X |  | X |  |  | X |  |  |  |  |  | **28.8** | 1.4 | 45.4 |
| 27 | NF1 | 26 | X | X | X | X |  |  |  |  |  |  |  |  |  |  |  |  | X |  |  |  |  |  | **19.8** | 1.9 | 35.3 |
| 28 | PTPN11 | 10 |  |  |  |  |  |  |  |  |  | X |  |  | X |  |  | X | X |  |  |  |  |  | **23.5** | 1.2 | 54.7 |
| 29 | NF1 | 12 | X | X |  |  |  |  |  |  |  |  |  |  |  | X |  |  | X |  |  |  |  |  | **20.1** | 0.8 | 43.4 |
| 30 | KRAS | 13 |  |  |  |  |  |  |  |  |  | X | X |  | X |  |  |  | X |  |  |  |  |  | **10.3** | 1.7 | 39.1 |
| 31 | PTPN11 | 9 |  |  |  |  |  |  |  |  |  |  | X |  | X |  |  |  | X |  |  |  |  |  | **20.9** | **3.5** | 48.5 |
| 32 | NF1 | 14 | X | X | X | X |  |  |  |  |  |  | X |  |  |  |  |  | X |  | X |  |  |  | **16.9** | **3.5** | 43.5 |
| 33 | NF1 | 20 | X | X |  |  |  |  |  |  |  |  |  | X |  |  |  |  | X |  |  |  |  |  | **12.3** | **3.6** | 32.6 |
| 34 | PTNP11 | 2 | X |  |  |  |  |  |  |  |  |  | X |  | X |  |  |  |  |  |  |  |  |  | **20.3** | 1.7 | 71.9 |
| 35 | PTEN | 12 |  |  |  |  |  | X |  | X | X |  |  | X |  |  |  |  |  |  |  |  | X |  | **30.3** | 2.0 | 41.0 |
| 36 | PTNP11 | 12 |  |  |  |  |  |  |  |  |  | X |  |  | X |  |  | X | X |  | X |  |  |  | **16.1** | 1.5 | 51.1 |
| 37 | NF1 | 10 | X | X |  | X |  |  |  |  |  |  | X |  |  |  |  | X | X |  |  |  |  |  | **17.1** | 1.8 | 57.3 |
| 38 | NF1 | 9 | X | X |  |  |  |  |  |  |  |  | X | X |  |  |  | X |  |  |  |  |  |  | **25.6** | 1.4 | 47.2 |
| 39 | PTPN11 | 21 |  |  |  |  |  |  |  |  |  | X |  |  | X |  |  | X | X |  |  | X |  |  | **27.7** | 2.0 | 10.7 |
| 40 | NF1 | 15 | X | X | X | X |  |  |  |  |  |  |  |  |  | X |  |  | X |  |  |  |  |  | **34.3** | **2.7** | 53.1 |
| 41 | Neg | 17 | X |  |  |  |  |  |  |  |  | X | X |  |  |  |  | X |  | X |  |  |  |  | **17.5** | 0.9 | 42.0 |
| 42 | PTEN | 3 |  |  |  |  |  |  |  | X | X |  | X |  |  |  |  |  |  |  |  |  |  |  | **22.7** | **2.9** | **39.8** |
| 43 | SHOC2 | 19 |  |  |  |  |  |  |  |  |  | X | X |  |  | X |  |  |  |  |  |  |  |  | **19.8** | 0.8 | 27.6 |
| 44 | NF1 | 3 | X | X |  |  |  |  |  |  |  |  |  |  | X |  |  |  |  | X | X |  |  |  | **28.7** | 2.2 | 68.6 |
| 45 | PTPN11 | 8 |  |  |  |  |  |  |  |  |  | X |  |  | X |  |  |  | X |  | X |  |  | X | **17.9** | **2.9** | **36.3** |
| 46 | NF1 | 12 | X | X | X | X |  |  |  |  |  |  | X | X |  |  |  |  |  |  | X |  |  |  | **9.8** | **3.3** | 44.2 |
| 47 | NF1 | 2 | X | X |  |  |  |  |  |  | X |  |  |  |  |  | X |  |  |  |  |  |  | X | 14.2 | 1.7 | 67.9 |

**Supplementary Table 3.** Clinical characteristics of the study population.

| **Phenotype** | **NF** | **NS** | **BRRS** |
| --- | --- | --- | --- |
| Number of patients  Sex | 33  16 Male | 11  6 Male | 3  3 Male |
| Genes (mutated) | NF1 27 (82%)  *Negative* 6 (18%) | PTPN11 8 (73%)  KRAS 1 (9%)  SHOC2 1 (9%)  *Negative* 1 (9%) | PTEN 3 (100%) |
| **Skin and soft tissue features**  Café-au-lait spot  Lentigines  Cutaneous neurofibromas  Plexiform neurofibromas  Xantogranulomas  Thin/absent hair  Genital lentiginosis  Lipomas | 33 (100%)  33 (100%)  32 (97%)  15 (45%)  10 (30%)  2 (6%)  0  0  0 | 3 (27%)  2 (18%)  0  0  0  0  1 (9%)  0  0 | 3 (100%)  0  0  0  0  0  0  2 (67%)  3 (100%) |
| **Short stature** requiring growth hormone therapy | 1 (3%) | 9 (82%) | 0 |
| **Developmental anomalies**  Cognitive impairment  Learning/behavioral problems | 11 (33%)  7 (21%)  7 (21%) | 6 (54%)  5 (45%)  1 (9%) | 3 (100%)  2 (66%)  1 (33%) |
| **Macrocephaly** | 10 (30%) | 0 | 3 (100%) |
| **Congenital heart defects** | 2 (6%) | 8 (73%) | 0 |
| **Ocular anomalies ***  Glaucoma  Exotropia  Peters’anomaly | 3 (9%)  1 (3%)  2 (6%)  0 | 5 (45%)  0  5 (45%)  1 (9%) | 0 |
| **Deafness** | 2 (6%) | 1 (9%) | 0 |
| **Genitourinary anomalies**  Renal anomalies  Cryptorchidism  Uterine anomalies  Gonadal dysgenesis | 5 (15%)  3 (9%)  1 (3%)  1 (3%)  1 (3%) | 2 (18%)  2 (18%)  1 (9%)  0  0 | 0 |
| **Skeletal anomalies**  Scoliosis  Limb length discrepancy  Chest wall anomalies | 9 (27%)  4 (12%)  3 (9%)  0 | 7 (63%)  4 (36%)  2 (18%)  3 (27%) | 0 |
| **Pulmonary anomalies** | 0 | 1 (9%) | 0 |
| **Feeding difficulties** | 0 | 1 | 0 |
| **SNC anomalies**  Optic pathway glioma  Chiari I malformation  Empty sella  Epilepsy | 6 (18%)  2 (6%)  2 (6%)  1 (3%)  1 (3%) | 2 (18%)  0  2 (18%)  0  0 | 0 |
| **Splenomegaly**** | 6 (18%) | 1 (9%) | 0 |
| **Lymphadenopathy** | 2 (6%) | 0 | 0 |
| **Autoimmunity**  Autoimmune thyroiditis  Alopecia areata | 1 (3%)  1 (3%)  0 | 0 | 1 (33%)  0  1 (33%) |
